# Circulating microRNA signature for the diagnosis of childhood dilated cardiomyopathy

**DOI:** 10.1038/s41598-017-19138-4

**Published:** 2018-01-15

**Authors:** Meng Jiao, Hong-Zhao You, Xin-Ying Yang, Hui Yuan, Yu-Lin Li, Wen-Xian Liu, Mei Jin, Jie Du

**Affiliations:** 1 Beijing Anzhen Hospital, Capital Medical University, Beijing, China; 2 Beijing Collaborative Innovation Centre for Cardiovascular Disorders, Beijing, 100029 China; 3 The Key Laboratory of Remodeling-Related Cardiovascular Diseases, Ministry of Education, Beijing Institute of Heart Lung and Blood Vessel Diseases, Beijing, 100029 China; 4 Department of Pediatric Heart Center, Beijing Anzhen Hospital, Capital Medical University, Beijing, 100029 China; 5 Department of Cardiology, Beijing Anzhen Hospital, Capital Medical University, Beijing, 100029 China; 6 Department of Clinical Laboratory, Beijing Anzhen Hospital, Capital Medical University, Beijing, China

## Abstract

Circulating miRNAs are proposed as a biomarker of heart disease. This study evaluated whether circulating miRNAs could be used as a biomarker for childhood dilated cardiomyopathy (CDCM). A total of 28 participants were enrolled in a discovery set, including patients with CDCM (n = 16) and healthy children (n = 12). The cardiac function of patients with CDCM was characterized by echocardiography and serum miRNA profiles of all participants were assessed by miRNA sequencing. After miRNA profiling, we quantitatively confirmed 148 regulated miRNAs in patients with CDCM compared with healthy subjects, and none were downregulated. Validation of candidate miRNAs was assessed by quantitative real-time polymerase chain reaction in other patients with CDCM (n = 30) and healthy controls (n = 16). A unique signature comprising mir-142-5p, mir-143-3p, mir-27b-3p, and mir-126-3p differentiated patients with CDCM from healthy subjects. Importantly, we observed an increase in mir-126-3p or let-7g in parallel with a robust decrease in the ejection fraction in patients with CDCM, which could differentiate heart failure patients from non-heart failure patients with CDCM. Moreover, mir-126-3p and let-7g were significantly negatively associated with the left ventricular ejection fraction. This study shows that a signature of four serum miRNAs may be a potential biomarker for diagnosing CDCM and assessing heart failure.

## Introduction

Dilated cardiomyopathy (DCM) is the most common childhood cardiomyopathy, with the incidence of newly diagnosed DCM reported to be approximately 0.53 cases per 100,000 population per year within the age range of 0 to 18 years in North America^1^. International registry data indicate that DCM is the most common reason for heart failure and cardiac transplantation in pediatric patients. The National Population-Based Study of Childhood Cardiomyopathy revealed that death or transplantation occurred in 26% of patients with childhood dilated cardiomyopathy (CDCM) within 1 year of diagnosis^2^.

DCM is characterized by ventricular chamber enlargement and systolic dysfunction. More than 50 gene mutations have been identified in DCM, including in the genes encoding cytoskeletal, nucleoskeletal, mitochondrial, and calcium-handling proteins^3^. miRNAs, as post-translational regulators of genes, modulate physiological and pathological processes^4^. miRNAs contribute to cardiac development, apoptosis, hypertrophy, fibrosis, and heart failure^5^. A previous study found a unique miRNA profile in left ventricles in pediatric patients different from that of adults with DCM^6^. In a murine phospholamban mutant model of dilated cardiomyopathy, miRNA microarray analysis revealed eight significantly downregulated pro-survival miRNAs in heart tissue that drive cardiomyocyte loss^7^. In an animal model of viral myocarditis, upregulation of miRNA21 promoted the progression of viral myocarditis to dilated cardiomyopathy^8^. In addition, long-term mir-669a therapy improved survival, ventricular dilation, and systolic function in Sgcb-null dystrophic mice. Understanding the signature in miRNA expression profiles in CDCM may facilitate the identification of a biomarker of its diagnosis/progression as well as new therapeutic targets, but few studies in CDCM have been performed.

Given the difficulties of tissue sampling in children, circulating miRNAs would be appealing as CDCM biomarkers, which would also provide biological insight into the mechanism behind this disease and its therapy targets. We thus aimed to characterize the profile of circulating miRNAs in CDCM and validate this expression pattern in additional subjects. Moreover, we assessed the sensitivity and specificity of miRNAs as a diagnostic biomarker and their correlation with the degree of heart function, as evaluated by transthoracic echocardiography. We found that eight circulating miRNAs were significantly increased in CDCM patients compared with their levels in matched subjectithout CDCM. Four miRNAs (mir-142-5p, mir-143-3p, mir-27b-3p, and mir-126-3p) served as sensitive and specific biomarkers in CDCM, which could potentially be used to diagnose this condition. Notably, only mir-126-3p was also correlated with heart failure.

## Results

### Clinical characteristics of patients

A total of 46 dilated cardiomyopathy patients were enrolled in this study, which included a discovery phase and a validation phase. The patients had been diagnosed by three pediatric cardiologists, based on echocardiography and clinical symptoms. 15 patients were in heart failure, as diagnosed by the Canadian Cardiovascular Society guidelines. In addition, 28 healthy age- and sex-matched subjects were enrolled in the study to serve as healthy controls, who had no family history of cardiovascular disease. Both CDCM patients and healthy children were recruited and enrolled from Beijing Anzhen Hospital, affiliated to Capital Medical University, China. The discovery phase included 12 healthy children and 16 CDCM patients, the clinical characteristics of whom are shown in Table 1. A total of 30 CDCM patients with/without heart failure and 16 healthy controls were used for subsequent validation of 11 selected miRNAs by quantitative real-time polymerase chain reaction (qRT-PCR) validation, for which the division into two subgroups (heart failure or non-heart failure) was based on the echocardiography results (shown in Table 2. The study design is shown in Supplementary Figure 1.

**Table 1:** Baseline characteristics of the Discovery Phase.

**Characteristics**	**Control**	**DCM**	**P value**
	**(n = 12)**	**(n = 16)**	
Age			
Mean ± SD,yrs	5.49 ± 3.69	5.27 ± 4.13	0.883
Median,yrs	5.3	4.59	0.798
Age group (n, %)			
<1yrs	1(8.33)	1(6.25)	0.683
1~10yrs	10(83.33)	12(75.00)	0.479
>10yrs	1(8.33)	3(18.75)	0.417
Male (n, %)	4(33.33)	6(37.50)	0.570
EF (%)	66.67 ± 11.80	31.50 ± 10.30	<0.001*
LVDD (mm)	34.25 ± 5.21	46.53 ± 8.45	<0.001*
FS (%)	33.28 ± 5.90	15.75 ± 5.15	<0.001*
Creatinine (umol/L)	39.00 ± 12.17	32.79 ± 12.09	0.192
Glucose (mmol/L)	4.72 ± 0.69	4.90 ± 0.66	0.493
Total glyceride (mmol/L)	0.71 ± 0.30	0.81 ± 0.30	0.407
Total cholesterol (mmol/L)	4.98 ± 1.33	4.10 ± 0.69	0.055
HDL cholesterol (mmol/L)	1.61 ± 0.43	1.34 ± 0.33	0.068
LDL cholesterol (mmol/L)	2.95 ± 1.13	2.47 ± 0.70	0.177

Values are mean ± SD or n(%). The p values are quoted for the Kruskal-Wallis or chi-square tests for continuous or categorical variables, respectively.

DCM: dilated cardiomyopathy; EF: Ejection Fractions; FS: fractionalshortening; HDL: high-density lipoprotein; LVEDD: left ventricular end-diastolic dimension; LDL: low-density lipoprotein.

**Table 2:** Clinical Characteristics of the Validation Phase Patients of subgroups varied according to ejection fraction, comparing to healthy controls.

**Characteristics**	**Control**	**DCM**	**DCM EF > 55%**	**DCM EF ≤ 55%**
	**(n = 16)**	**(n = 30)**	**(n = 15)**	**(n = 15)**
Age				
Mean ± SD,yrs	5.97 ± 4.41	4.15 ± 3.85	3.97 ± 2.79	4.33 ± 4.77
Median,yrs	4.5	3	3	2
Age group (n, %)				
<1 yrs	2(12.50)	9(30.00)	3(20.00)	6(40.00)
1~10yrs	10(62.50)	18(60.00)	12(80.00)	6(40.00)
>10yrs	4(25.00)	3(10.00)	0	3(20.00)
Male (n,%)	9(56.25)	13(43.33)	7(46.67)	6(40.00)
EF (%)	65.19 ± 13.58	50.20 ± 17.24*	66.53 ± 2.95	33.87 ± 5.91*#
LVDD (mm)	35.06 ± 5.13	44.63 ± 12.69*	39.53 ± 8.41	49.73 ± 14.41*#
FS (%)	32.56 ± 6.78	25.10 ± 8.62*	33.27 ± 1.47	16.93 ± 2.95*#
Creatinine(umol/L)	34.24 ± 10.97	30.17 ± 13.00	28.37 ± 11.17	31.97 ± 14.78
Glucose(mmol/L)	4.60 ± 0.52	4.82 ± 0.53	4.69 ± 0.55	4.96 ± 0.49
Total glyceride(mmol/L)	0.90 ± 0.80	0.88 ± 0.43	1.03 ± 0.41	0.73 ± 0.40
Total cholesterol (mmol/L)	4.26 ± 0.81	4.01 ± 0.84	4.03 ± 0.87	3.99 ± 0.85
HDL cholesterol (mmol/L)	1.32 ± 0.42	1.37 ± 0.34	1.39 ± 0.40	1.36 ± 0.26
LDL cholesterol (mmol/L)	2.53 ± 0.65	2.25 ± 0.74	2.20 ± 0.78	2.31 ± 0.72

Values are mean ± SD or n(%). The p values are quoted for the Kruskal-Wallis or chi-square tests for continuous or categorical variables, respectively.

Comparative with healthy controls, *P < 0.05; Comparative with DCM EF ≤ 55%, ^#^*P* < 0.05.

DCM: dilated cardiomyopathy; EF: Ejection Fractions; FS: fractionalshortening; HDL: high-density lipoprotein; LVEDD: left ventricular end-diastolic dimension; LDL: low-density lipoprotein.

### Identification of miRNAs differentially expressed in CDCM patients using next-generation sequencing analysis

To identify miRNA that are differentially expressed in CDCM and control, we performed miRNA sequencing on RNA from the serum of 16 CDCM and 12 health controls.

**Figure 1:**
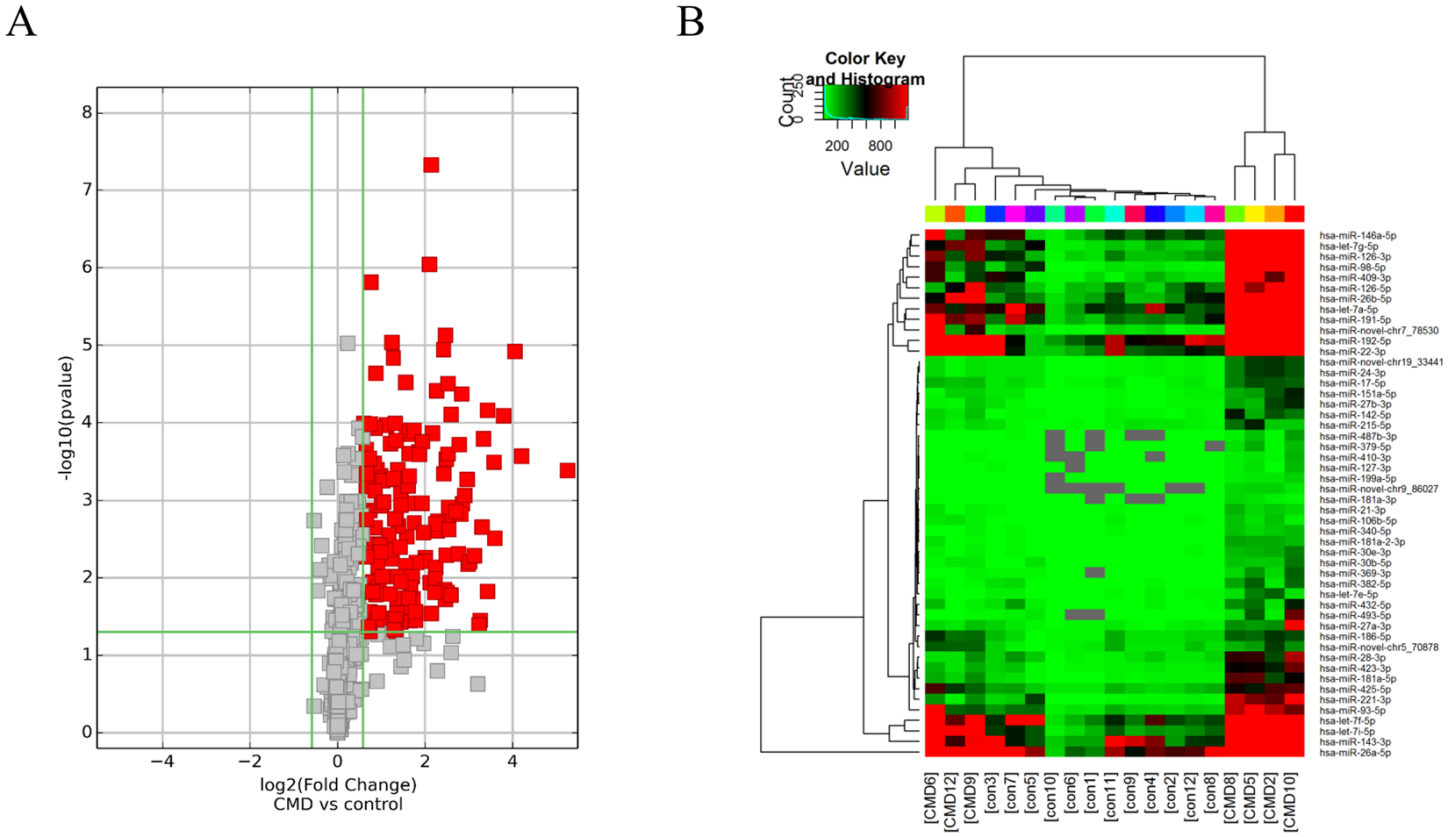
Overview of miRNAs Sequencing Data. (**A**)Volcano plot of differentially expressed miRNAs between DCM patients and healthy controls. Volcano plot of detectable genome-wide miRNA profiles in differentiating 16 DCM cases and 12 controls. The x-axis shows the log2 foldchange in circulating miRNAs’ expression between DCM cases and controls, while the y-axis shows the −log10 of p value for each miRNA. (**B**) Heat map of differentially expressed miRNAs between DCM patients and healthy controls. The result of unsupervised hierarchical clustering of circulating miRNAs from DCM patients and healthy control. The color scale shown at the top illustrates the relative expression level of a miRNA, comparing the DCM patients group clusters with the CON group. Red color represents an expression level above mean and green color represents expression lower than the mean.

A total of 1675 miRNAs were identified in both groups. We performed filtering between the CDCM patients and healthy controls, using the following criteria: fold change ≥2.0 (or ≤0.5) and P-value ≤ 0.05. A hierarchical clustering of differential miRNA was shown in Fig. 1A, including 148 upregulated miRNAs and no downregulated miRNA.

Unsupervised hierarchical cluster analysis further revealed that the serum sample of CDCM patients could be distinguished clearly from that of healthy children based on their pattern of significantly higher expression of certain miRNAs. Among the 148 miRNAs analyzed, and the TOP 50 miRNAs were as shown in Fig. 1B. Eleven candidate miRNAs (shown in Supplementary Table 1) that were particularly abundant and for which the difference in expression in serum was highly significant were selected to determine the validity of the previously obtained results.

### Kyoto Encyclopedia of Genes and Genomes (KEGG) pathway analyses

A total of 10,543 target genes were predicted for the 148 differentially expressed miRNAs; the number of regions of overlap among the three different databases was 73. Gene Ontology enrichment analysis was used on the target gene candidates of the top 10 differentially expressed miRNAs. The predicted target genes of the differentially expressed miRNAs were further classified to annotate the pathways in which they are involved, using KEGG pathway annotation. A total of 24 associated biological pathways were identified. The top 10 pathways are shown in Supplementary Figure 2. Some of these pathways, such as the MAPK pathway, the FoxO signaling pathway, and the Erb signaling pathway, were shown to be involved in cardiac fibrosis and apoptosis^9–11^. Our data demonstrate the broad range of target genes for those differentially expressed miRNAs potentially involved in the pathological process of DCM.

### Validation of differentially expressed miRNAs using qRT-PCR

According to the data from miRNA sequencing described previously, we selected 11 miRNAs that were significantly upregulated in CDCM patients and highly abundant in serum, namely, miR-26a5p, let-7g-5p, miR-143-3p, miR-126-3p, miR-24-3p, let-7f-5p, let-7i-5p, miR-142-5p, miR-27a-3p, miR-27b-3p and miR-98-5p. To validate the significant changes in miRNA expression, these miRNAs were assessed by qRT-PCR. Independent sets of CDCM patients (n = 30) and control healthy subjects (n = 16) were used in this validation phase, the baseline characteristics of whom are shown in Table 2. Of the candidate miRNAs, let-7f-5p, let-7g-5p, miR-142-5p, miR-126-3p, miR 143-3p, miR-26a-5p, miR-27a-3p, and miR-27b-3p showed statistically significant upregulation in CDCM patients compared with that in controls; however, there were no significant differences for miR-98-5p and miR-24-3p. Given their significantly higher levels in patient serum, these eight miRNAs may have potential as diagnostic biomarkers.

### Diagnostic value of miRNAs for CDCM

In the validation phase, mean age and gender were similar between the CDCM cases and controls, as shown in Table 2.

**Figure 2:**
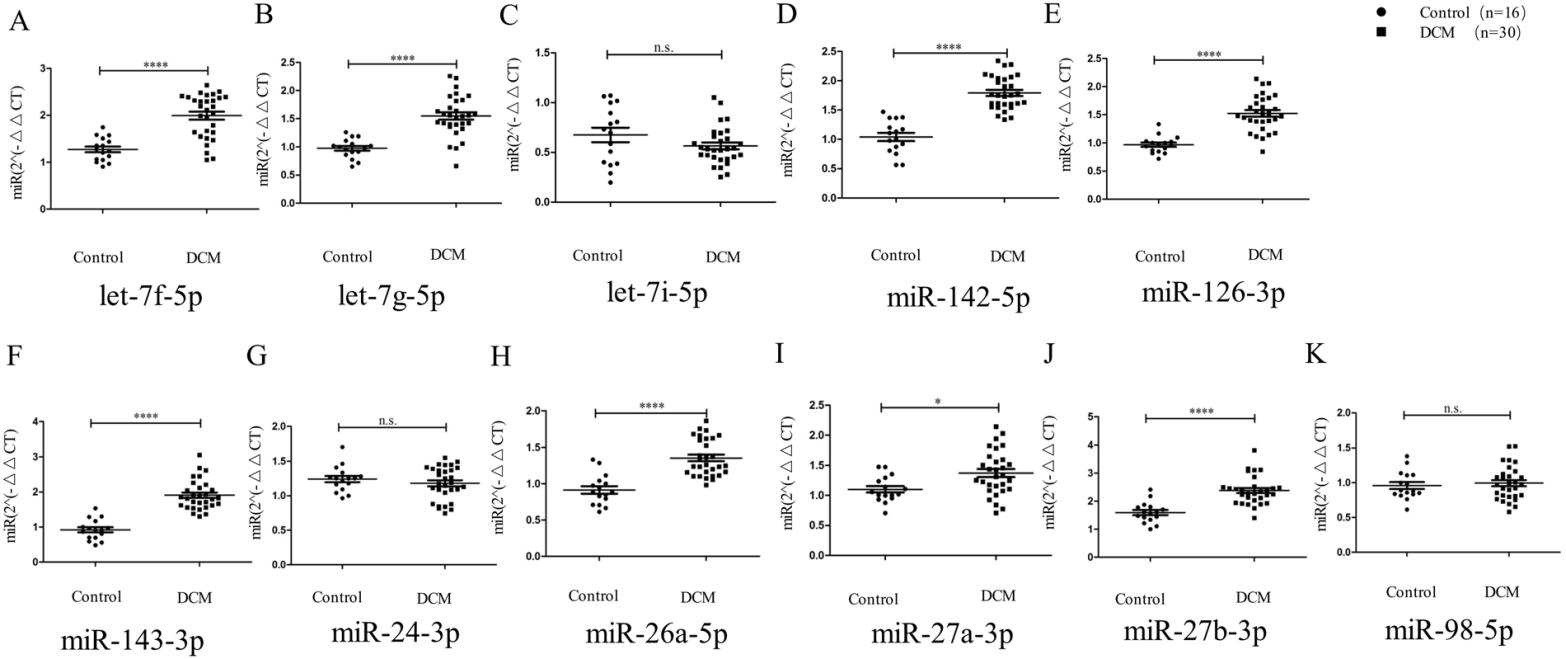
Quantitative RT-PCR analysis of differentially expressed miRNAs in serum. Comparative analysis of expression levels of (**A**) let-7f-5p, (**B**) let-7g-5p (**C**) let-7i-5p (**D**) miR-142-5p (**D**) miR-1425p (**E**) miR-126-3p (**F**) miR-143-3p (**G**) miR-24-5p (**H**) miR-26a-3p (**I**) miR-27a-3p (**J**) miR-27b3p (**K**) miR-98-5p by quantitative RT-PCR analysis in an validation phase. ****P < 0.0001; ***P, < 0.001; **P < 0.01; *0.01 < P < 0.05.

**Figure 3:**
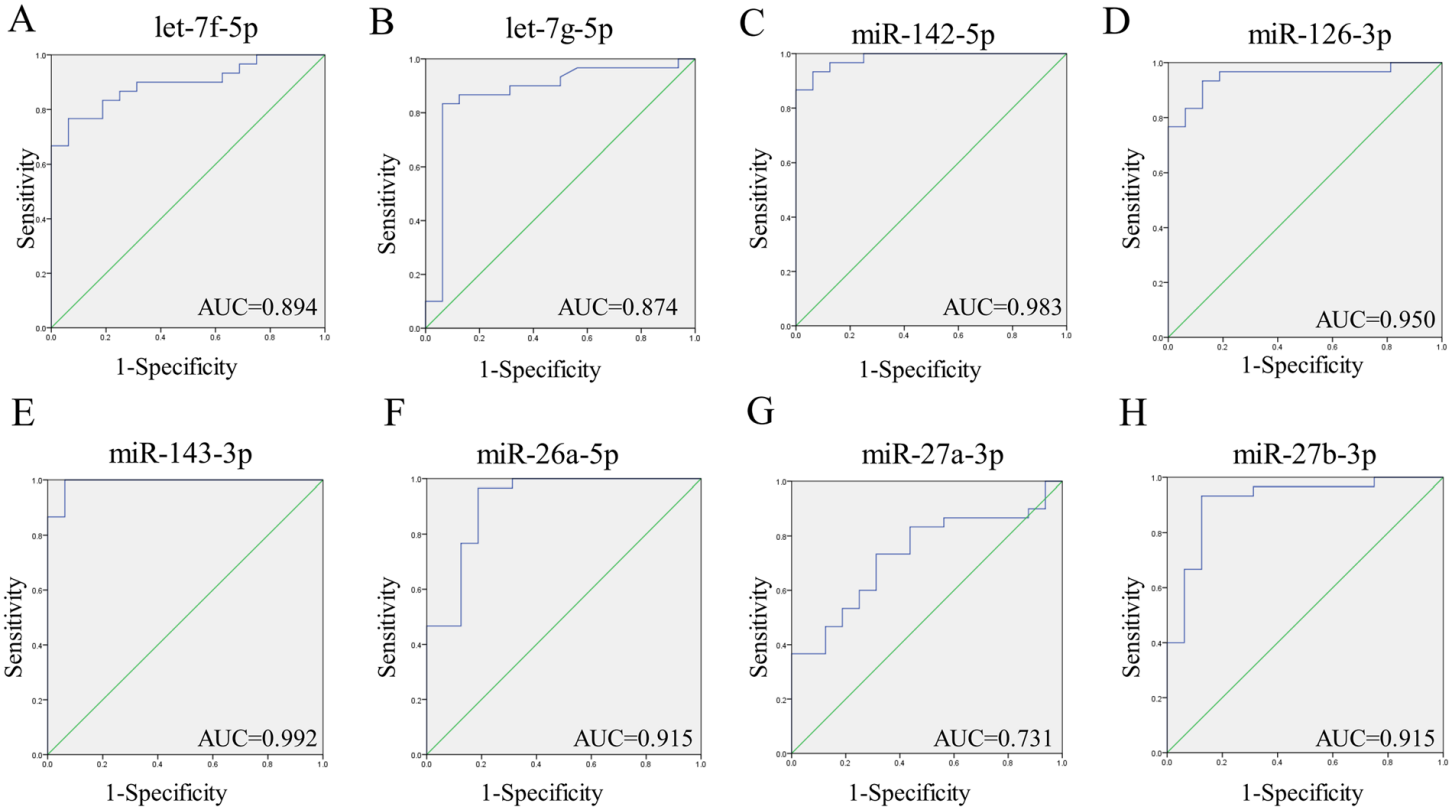
Receiver operating characteristic(ROC) curve for single circulating miRNA for the diagnosis DCM.

To discriminate between the patients with CDCM and the healthy controls (diagnostic value), the receiver-operating characteristic curves were plotted for every single miRNA in validation of qRTPCR that was expressed at a significantly higher level in patient serum (Fig. 2A–K). The areas under the curves (AUCs) ranged from 0.731 to 0.992, and almost all of them were higher than 0.9 (let-7g5p, miR-142-5p, miR-126-3p, miR-143-3p, miR-26a-5p, and miR-27b-3p), suggesting that these circulating miRNAs may be useful for CDCM detection and diagnosis(Fig. 3A–H and Supplementary Table 2).

### miRNAs were associated with heart dysfunction in CDCM

**Figure 4:**
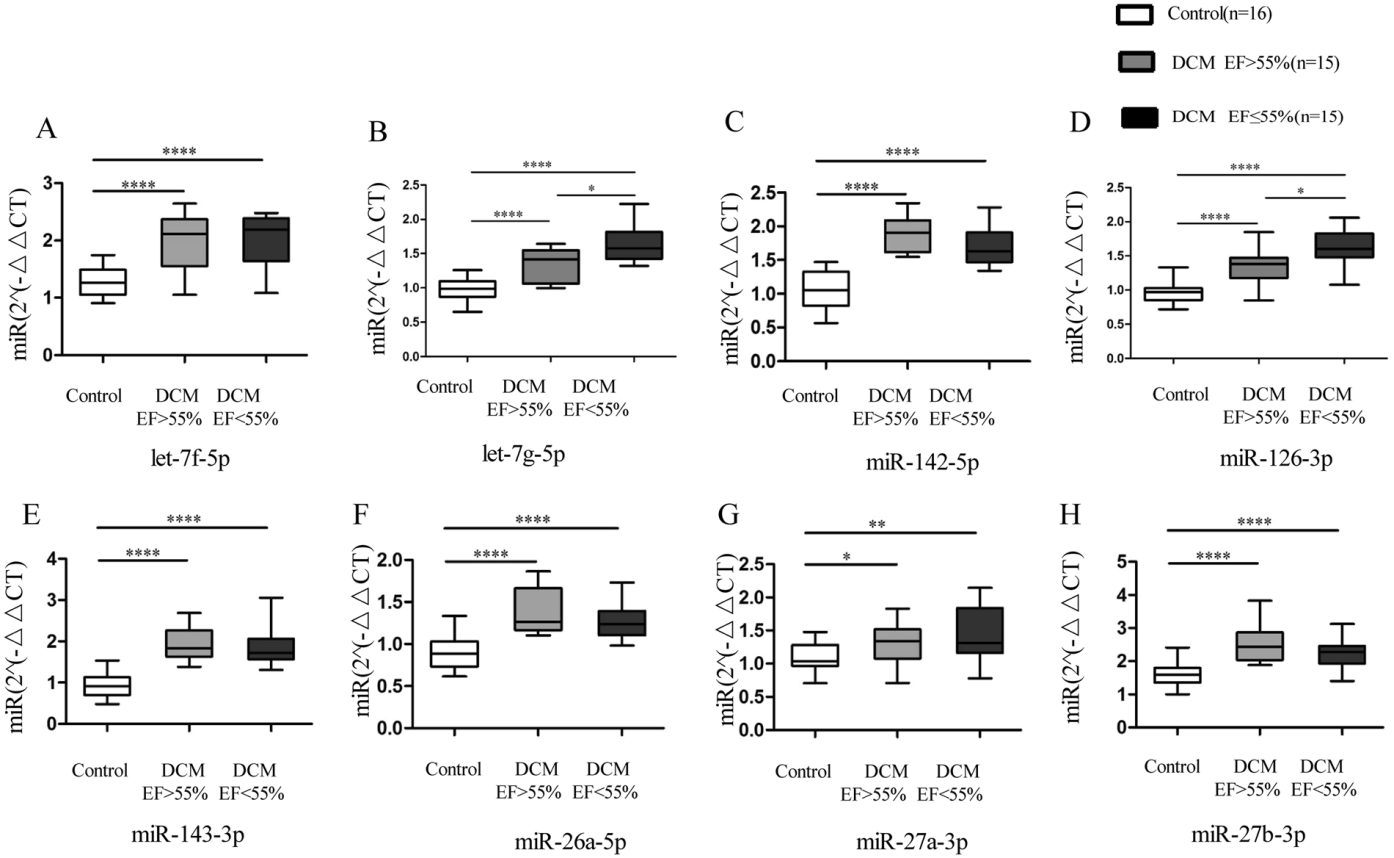
Comparison of miRNAs in DCM patients with different cardiac function. Comparative analysis of expression levels for selected microRNA candidates for DCM EF > 55%, DCM EF ≤ 55% and healthy controls; ****P < 0.0001; ***P < 0.001; **P < 0.01; *0.01 < P < 0.05.

**Figure 5:**
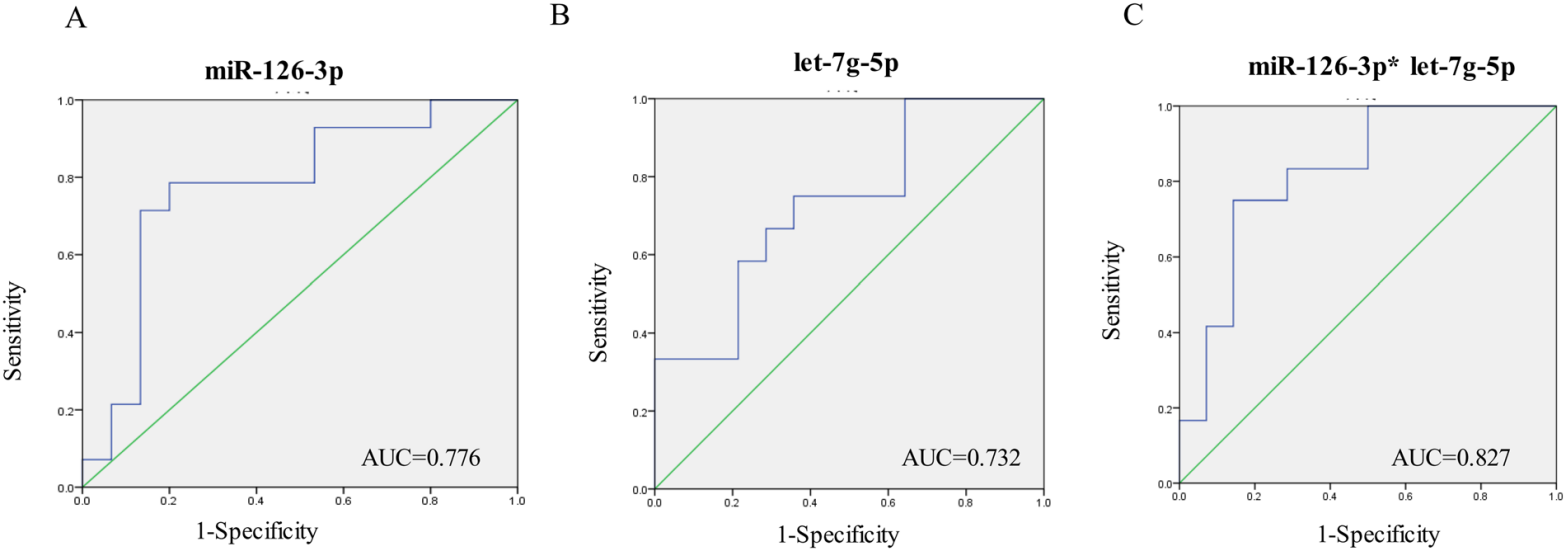
ROC plots of the individual and combined 2 miRNAs differentially expressed between the CDCM patients with heart failure and non-Heart failure.

**Figure 6:**
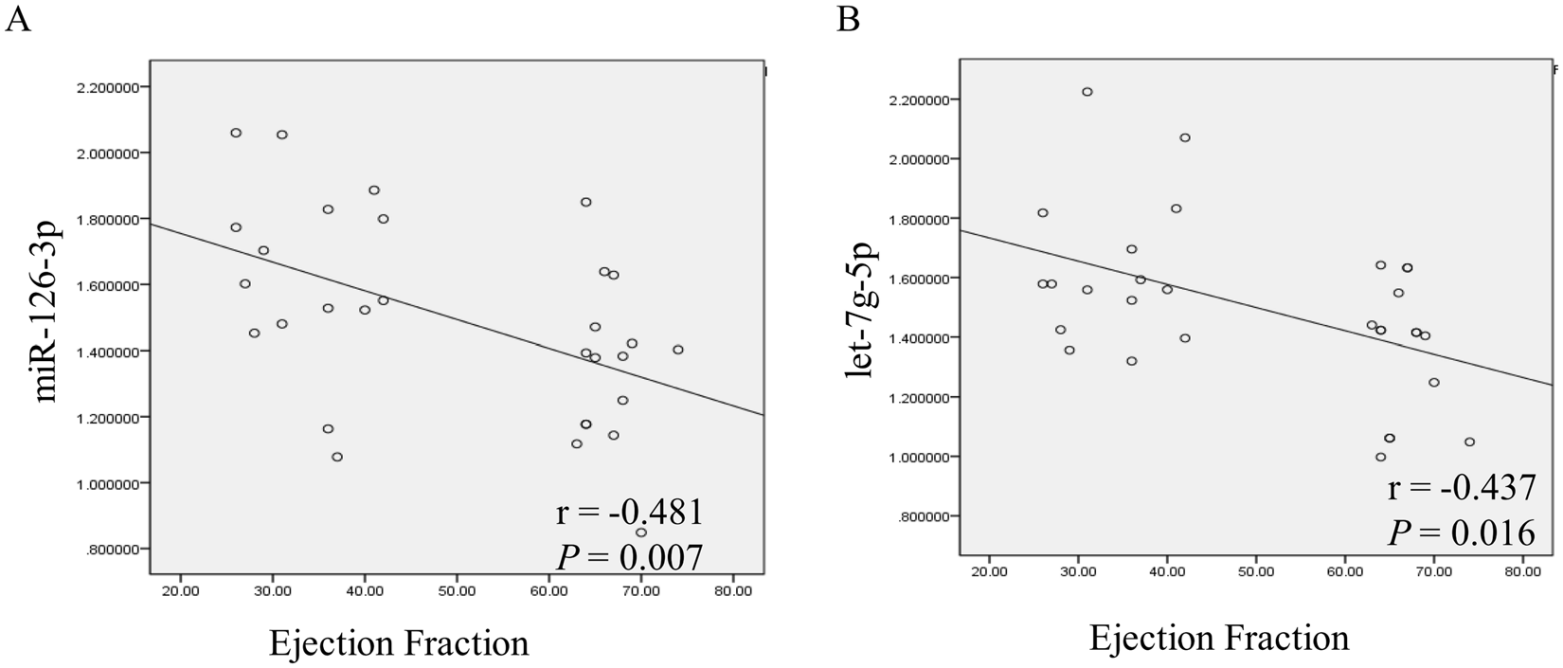
Linear Regression Analysis of ejection fraction and microRNA in DCM patients. Linear Regression Analysis of EF and MicroRNA in DCM patients.

To evaluate the role of miRNAs in the classification of CDCM patients, 30 CDCM patients were divided into two subgroups according to ejection fraction (EF) in the conventional transthoracic echocardiography (non-heart failure, EF >55%; heart failure, EF ≤55%). There was no significant difference in clinical characteristics, including gender, age, and ultrasound results, except for ejection fraction and left ventricular end diastolic diameter. The relationship between the expression level of circulating miRNAs and cardiac function was further studied. In 8 selected miRNAs, mir126-3p and let-7g-5p expression significantly increased in the CDCM patients with heart failure compared with that in the non-heart failure CDCM patients (p < 0.05) (Fig. 4A–H), suggesting that circulating miRNAs may be associated with the progression of heart failure in CDCM patients. The areas under the curves (AUCs) of miR-126-3p and let-7g-5p were 0.782 and 0.760, respectively. Interestingly, a combination of these two miRNAs increased the AUC to 0.849 (Fig. 5A–C). We also examined the relationship of the concentration of mir-126-3p with the ejection fraction as a measure of heart function. As illustrated in Fig. 6, there was a significant inverse correlation of miR-1263p or let-7g with ejection fraction (r = −0.481, p = 0.007; r = −0.437, p = 0.016). These results suggested the potential of miR-126-3p and let-7g was a novel biomarker to predict cardiac dysfunction in pediatric DCM patients.

## Discussion

In the present study, a set of miRNA biomarkers were discovered and validated by the evaluation of serum miRNA profiles from two experimental sample sets. The first set was profiled using miRNA sequencing to identify differentially expressed miRNAs specific to DCM in children. A quantitative RT-PCR assay was then performed to validate the candidate differentially expressed miRNAs in the second sample set. The main findings of this study can be summarized as follows: (i) a substantial difference in miRNA expression between CDCM and healthy controls; (ii) a novel signature of four circulating miRNAs (mir-142-5p, mir-143-3p, mir-27b-3p, and mir-126-3p) allowing CDCM patients to be distinguished from healthy controls; and (iii) only mir-126-3p negatively correlated with heart systolic dysfunction.

An increasing amount of evidence indicates that miRNAs play a crucial role in idiopathic dilated cardiomyopathy. First, miRNAs may be involved in the mechanism regulating the development of DCM. Bioinformatic analysis revealed that the downregulation of key pro-survival miRNAs promoted apoptotic signaling and heart decompensation^7^. Using mdx mice as a model of Duchenne muscular dystrophy-induced cardiomyopathy, another previous study found that the downregulation of mir448 promoted cellular oxidative stress, which subsequently triggered cardiomyocyte death and cardiomyopathy progression^12^. Cardiomyocyte-specific miR-30C overexpression causes DCM by inhibiting mitochondrial oxidative phosphorylation in complexes III and IV^13^. Biomarkers have taken on an important role in the diagnosis and prognosis of heart failure in adults^14^. Second, serum miRNAs are remarkably stable. A previous study reported that serum miRNAs (mir-3135b, mir-3908, and mir-5571-5p) could be used as diagnostic biomarkers for adult DCM^15^. The levels of serum mir5571-5p significantly correlated with the New York Heart Association classification. However, little is known about the profile of circulating miRNAs and their diagnostic value in pediatric DCM. So far, Brian’s group found four miRNAs (mir-155, mir-636, mir-646 and mir-639) are specific to CDCM who have the potential to recover^16^. There is thus a need for involvement of miRNAs in pediatric DCM patients to be more clearly defined, for their potential application in assisted diagnosis and risk stratification. Using miRNA sequencing, we found 148 upregulated miRNAs in CDCM, but no downregulated ones (Fig. 2). qRT-PCR assay showed that miRNA alterations were well correlated with the findings from miRNA sequencing in independent sets. GO enrichment analysis based on the target genes of the differentially expressed miRNAs showed that the top 10 biological processes involved metabolic processes. Adult DCM patients exhibited decreased fatty acid metabolism and increased myocardial glucose metabolism^17^. The specific miRNA profile in CDCM patients may reflect abnormalities in heart metabolism. At present, there are no biomarkers to differentiate DCM from healthy subjects. In our study, we identified for the first time a unique signature of differentially expressed miRNAs that can distinguish CDCM patients from healthy children.

The levels of eight miRNAs (let-7f, let-7g, mir-142-5p, mir-143-3p, mir-26a, mir-27a-3p, mir-27b-3p, and mir-126-3p) were significantly increased in the serum of CDCM patients. Mir-27a, mir26a, mir-143, and mir-126-3p were significantly increased in HCM serum. Only mir-27a was correlated with hypertrophy^18^. In another study, circulating mir-26a was found to be significantly increased in Takotsubo cardiomyopathy patients compared with its levels in healthy subjects, distinguishing those with Takotsubo cardiomyopathy from STEMI patients^19^. Members of the let-7 family drive embryonic stem cell-derived cardiomyocyte maturtation by downregulation of the PI3K/AKT protein kinase/insulin pathway^20^. In addition, mir-142 was found to be suppressed in the heart of a hypertrophic model and in human cardiomyopathic heart. Forced overexpression of mir-142 induced extensive cardiomyocyte death and cardiac dysfunction^21^. However, to our knowledge, circulating mir-142, let-7f, and let-7g have not previously been linked to DCM. Furthermore, four of these eight miRNAs (mir-126-3p, mir143-3p, mir-26a, and mir-142-5p) had an ROC curve that distinguished the CDCM group from the healthy group (AUC > 0.9). mir-142-5p and mir-143-3p yielded sensitivity of 93.3% and 100% and specificity of 93.8% and 93.8% for CDCM compared with healthy controls, respectively.

Left systolic function is associated with clinical outcomes in dilated cardiomyopathy patients^22^. To explore the correlation between miRNA expression and heart function, CDCM was divided into two subgroups based on EF (non-heart failure, EF > 55%; heart failure, EF < 55%). Mir-126-3p and let-7g exhibited a significant difference in expression between the non-heart failure and heart failure groups. However, we found an increase of mir-126-3p and let-7g in parallel with a robust decrease of EF in CDCM patients (p < 0.05). mir-126, as an important regulator of endothelial cell homeostasis, holds promise as a prognostic biomarker of vascular damage and endothelial dysfunction. Recent basic and clinical research has demonstrated its important role in heart failure. In contrast with our results, in atrial fibrillation patients, mir-126 expression was positively correlated with LVEF and negatively correlated with the N-terminal pro-brain natriuretic peptide levels^23^. However, in coronary heart disease patients, a positive association between circulating mir-126 and fatal myocardial infarction was identified^24^. Genome-wide profiling of microRNAs revealed that the expression of let-7 family was upregulated in heart failure tissue from dilated cardiomyopathy and ischemic cardiomyopathy patients compared with normal heart tissue^25^. Cardiomyocyte death/regeneration and metabolic disorder promoted development of heart failure. Let-7 family regulated cardiomyocyte regeneration, cell survival, metabolic control and glucose utilization^26^. The clinical study found the let-7g combined with other 4 miRNAs predict future fatal myocardial infarction in health participants^27^. In the present study, a negative correlation between mir126 or let-7g and LVEF was found, suggesting mir-126 and let-7g as a potential biomarker for the assessment of heart failure in CDCM. But further study is needed to determine whether mir126 and let-7g may participate in the pathogenesis of DCM in children.

However, the present study has certain limitations. First, the current lack of knowledge about the biology of miRNAs hinders our ability to assess the level of circulating miRNAs accurately. In addition, the sample size in this single-center study was small. Fewer controls were enrolled in the validation phase, because recruiting the healthy children is challenging. It is believed that the diagnostic value of miRNAs should be further validated in a multi-center study. Moreover, this study involved a cross-sectional study and there were no long-term follow-up data on the prognosis of CDCM. Future studies are needed to overcome these limitations.

In summary, the profile of four circulating miRNAs was found to distinguish CDCM patients from healthy children. Mir-126-3p was also shown to be negatively correlated with LVEF.

## Materials and Methods

### Patients

A total of 46 patients were enrolled in the study at Anzhen Hospital from November 2015 to May 2016. DCM patients, whose age from 9 months to 13 years old, were all diagnosed by clinical symptoms and echocardiography. 28 healthy children were also enrolled at the hospital as health control, with matched age and gender. Characteristics of the study including discovery phase and validation phase are given in Tables 1 and 2.

All ultrasound examinations were performed using a commercially available echocardiographic machine with an S3 transducer (Vivid Seven System, GE Healthcare, Horten, Norway) when DCM and healthy subjects(CON) finished the collection of blood sample. The LV end-diastolic volume (EDV), end-systolic volume (ESV), and EF were calculated using biplane Simpson’s methods from apical four and two-chamber views. The diagnosis of heart failure was based on clinical presentation and echocardiographic results according to 2013 Canadian Cardiovascular Society guideline^28^. The study protocol was approved by the Medical Ethics Committee of Beijing Anzhen Hospital affiliated to Capital Medical University and carried out according to the principles of the Declaration of Helsinki. All informed consents were obtained from children’s parents or guardians. The general characteristics of participants were shown in Supplementary Table 3, and more details are shown online (URL: http://www.clinicaltrials.gov/, unique identifier: NCT03076580).

### Serum collection and storage

All of blood samples were collected from participant and drawn into a sterile polyolefin resin tube with coagulant, then centrifuged at 3000 rpm for 10 minutes in the clinical lab. The supernatant serum was quickly removed, aliquoted in the RNase-free microfuge tube and stored at −80 centigrade.

### RNA preparation and sequencing

Total serum RNA were isolated from the discovery set of serum samples was extracted using mirVana miRNA isolation kit following the manufacture’s protocol (Ambion, Thermo Fisher Scientific Inc, Waltham, MA USA). Each RNA sample was quantified with a spectrophotometer (NanoDrop 1000, Thermo Scientific, Wilmington, Delaware). Total RNA was used to prepare the miRNA sequencing library, which included the following steps: 1) 3′-adaptor ligation; 2) 5′adaptor ligation; 3) cDNA synthesis; 4) PCR amplification; 5) size selection of ~135–155 bp PCR amplified fragments (corresponding to ~15–35 nt small RNAs).The libraries were denatured as single-stranded DNA molecules, captured on Illumina flow cells (Illumina, Inc, San Diego, CA, USA), amplified *in situ* as clusters and finally sequenced for 36 cycles on Illumina NextSeq. 500 per the manufacturer’s instructions.

### Target genes analysis of the validated miRNAs

To better understand the biological function of the selected 11 differentially expressed miRNAs (miR-26a-5p, let-7g-5p, miR-143-3p,miR-126-3p, miR-24-3p, let-7f-5p, let-7i-5p, miR-142-5p, miR-27a-3p, miR-27b-3p and miR-98-5p), their putative target genes were predicted using three database miRanda database (http://www.microrna.org/ microrna/home.do), Microcosm Targets Version5 (formerly miRBase Targets, the website is (http://www.ebi.ac.uk/enright-srv/microcosm/htdocs/targets/v5/) and Targetscan version 6.2 (http://www.targetscan.org/vert_60/).

To improve prediction accuracy, we choose the putative target gene identified in all three database. To gain insight into the biological functions of the differential miRNAs., the pathways of target genes were analyzed, using KEGG (http://www.genome.jp/)

### Validation of candidate miRNAs expression with quantitative real time RT-PCR (qRT-PCR)

Total serum RNA was isolated from the validation set of serum samplesWe spiked 100 pM of synthetic cel-mir-40-3p into serum RNA.Isolated RNA wasconverted to complementary DNA (cDNA) with TaqMan miRNA Reverse Transcription Kit (Life Technologies, Foster City, CA). TaqMan (miR-26a-5p, let-7g-5p, miR-143-3p, miR-126-3p, miR-24-3p, let-7f-5p, let-7i-5p, miR 142-5p, miR-27a-3p, miR-27b-3p and miR-98-5p), RT primers, 2ng of total RNA from each sample was reverse-transcribles following the manufacture’s protocol (Applied Biosystems, Foster City, California). qRT-PCR reaction was conducted in 96-well plates with RT product with TaqMan PCR master mix and TaqMan probes for each miRNA by ABI Prism Model 7900 HT instrument. Quantitative real-time polymerase chain reactions were performed in duplicate for all samples. The data analyzed using the comparative Ct method. miRNA expression levels were normalized to a nonendogenous synthetic miRNA. The PCR primers for miRNA were shown in supplementary Table 4.

### Statistical analysis

Values are expressed as mean + SEM or median. Shapiro–Wilk and Kolomogorov–Smirnov tests were used to test for non-Gaussian distribution. Student’s two-sided t-test or one-way ANOVA, followed by Bonferroni’s multiple comparison test as a post hoc analysis, was used for normally distributed values. For variables w.ithout normal distribution, Mann–Whitney U test or Kruskal– Wallis test was performed. Receiver operating characteristic curves were analysed to assess specificity and sensitivity of single-plasma miRNAs and their combination using multiple logistic regression analysis. The optimal diagnostic point of the signature was assessed at cut-off values with the largest Youden’ s index (sensitivity + specificity −1). To investigate the clinical impact of our signature miRNAs, we have chosen the likelihood ratio (LR) in addition to classical test parameters— sensitivity and specificity. Odds ratio was calculated by logistic regression analysis. Linear regression analysis was performed to determine the relationships between Ejection Fractions (EF) and miR-126-3p the using spearman rank correlation. Statistical analyses were performed using SAS version 9.3 (SAS Institute Inc., Cary, North Carolina), IBM SPSS Statistics 20 (SPSS,Inc., Chicago, IL, USA), and GraphPad Prism 5. The following values were considered significant: *p* < 0.0001(****); *p* < 0.001(***); *p* < 0.01(**); 0.01 < *p* < 0.05(*).

## Electronic supplementary material

Supplementary Material
