# Supplementary material for: Circulating microRNA signature for the diagnosis of childhood dilated cardiomyopathy
Source: Sci Rep. 2018 Jan 15;8:724. doi: 10.1038/s41598-017-19138-4 (PMC5768721; doi:10.1038/s41598-017-19138-4)

Circulating microRNA signature for the diagnosis of childhood dilated cardiomyopathy

Meng Jiao[1,2*], Hong-Zhao You[1*], Xin-Ying Yang[1], Hui Yuan[4], Yu-Lin Li[1], Wen-Xian Liu[3], Mei Jin[2┼], Jie Du[1┼]

[1] Beijing Anzhen Hospital, Capital Medical University, Beijing, China; Beijing Institute of Heart, Lung, and Blood Vessel Diseases, Beijing, China; The Key Laboratory of Remodeling-Related Cardiovascular Diseases, Ministry of Education, Beijing, China

[2] Department of Pediatric Heart Center, Beijing Anzhen Hospital, Capital Medical University, Beijing 100029, China.

[3]Department of Cardiology, Beijing Anzhen Hospital, Capital Medical University, Beijing 100029, China.

[4]Department of Clinical Laboratory, Beijing Anzhen Hospital, Capital Medical University, Beijing, China.

* Meng Jiao and Hong-Zhao You contributed equally to this work

┼Please address the correspondence to:

Dr. Jie Du, E-mail: jiedu@ccmu.edu.cn；

Dr. Mei Jin, E-mail: Jinmei61@sohu.com.

Supplementary Matetrial

Supplementary Table 1 Validation of selected miRNA by qPCR
Supplementary Table 2 Receiver operating characteristic curves.
Supplementary Table 3 Baseline characteristics of participants in the whole study
Supplementary Table 4 qPCR Primers used for detecting miRNAs expression

Supplementary Figure 1. Study Design. Summary of workflow showing numbers of both DCM patients and healthy controls recruited in discovery and validation phases.

Supplementary Figure 2. KEGG pathway analysis and classification for target genes of differentially expressed miRNAs. The figure shows KEGG enrichment for the target genes. The Y-axis pathways of target gene productions involved in, and the X-axis shows the −log10 of p value for each.

Supplementary Table 1 Validation of selected miRNA by qPCR

**microRNA**	**Accession Number**	**DCM Group**	**CON Group**	**Fold-change （DCM/CON）**	**P-value**
**miR-98-5p**	**MI0000100**	**2305.1**	**124.75**	**18.47795591**	**<0.01**
**let-7i-5p**	**MI0000434**	**5015.8**	**302.67**	**16.57184298**	**<0.01**
**miR-26a-5p**	**MI0000083**	**11705**	**844.42**	**13.86112208**	**<0.01**
**let-7g-5p**	**MI0000433**	**2382.7**	**199.83**	**11.92339604**	**<0.01**
**miR-126-3p**	**MI0000471**	**2506.7**	**232.83**	**10.76603402**	**<0.01**
**miR-142-5p**	**MI0000458**	**385.25**	**35.833**	**10.75116279**	**0.015**
**let-7f-5p**	**MI0000067**	**5367.7**	**532.17**	**10.08647286**	**<0.01**
**miR-27b-3p**	**MI0000440**	**251.75**	**36.667**	**6.865846674**	**<0.01**
**miR-24-3p**	**MI0000080**	**301.44**	**46.167**	**6.529332129**	**<0.01**
**miR-27a-3p**	**MI0000085**	**333.88**	**57.167**	**5.840344954**	**<0.01**
**miR-143-3p**	**MI0000459**	**4384.4**	**783.42**	**5.596558876**	**<0.01**

Supplementary Table 2. Receiver operating characteristic curves

	AUC	95%CI	OR Per 0.1SD	95%CI	Cut-off	Sensitivity (%)	95%CI	Specificity (%)	95%CI	LR+	LR-
**DCM vs. healthy**											
**let-7f-5p**	0.89	0.804 - 0.890	1.26	1.10-1.49	1.61	0.77	57.72-90.07	93.8	69.77 - 99.84	12.27	0.25
**let-7g-5p**	0.92	0.844 - 0.924	1.34	1.12-1.59	1.29	0.83	65.28-94.36	100	79.41 - 100.0	-	0.17
**miR-142-5p**	0.98	0.956 - 0.983	2.25	1.12-4.52	1.39	0.93	77.93-99.18	93.8	69.77 - 99.84	14.93	0.07
**miR-126-3p**	0.95	0.877 - 0.950	1.47	1.16-1.86	1.11	0.93	77.93-99.18	87.5	61.65 - 98.45	7.47	0.08
**miR-143-3p**	0.99	0.973 - 0.992	2.11	1.15-3.88	1.30	1	88.43-100.0	93.8	69.77 - 99.84	16.00	0.00
**miR-26a-5p**	0.92	0.820 - 0.915	1.39	1.13-1.71	1.05	0.97	82.78-99.92	81.3	54.35 - 95.95	5.16	0.04
**miR-27a-3p**	0.73	0.585 - 0.731	1.11	1.02-1.20	1.15	0.73	54.11-87.72	68.8	41.34 - 88.98	2.35	0.39
**miR-27b-3p**	0.92	0.823 - 0.915	1.34	1.13-1.59	1.88	0.93	77.93-99.18	87.5	61.65 - 98.45	7.47	0.08

Supplementary Table 3. Baseline characteristics of participants in the whole study

**Characteristics**	**Control (n=28)**	**DCM (n=46)**	**P value**
**Age**			
**Mean±SD,yrs**	5.77±4.05	4.54±3.94	0.203
**Median,yrs**	5.00	3.00	0.130
**Age group(n,%)**			
**＜1yrs**	3(10.71)	10(21.74)	0.347
**1～10yrs**	20(71.43)	30(65.22)	0.580
**>10yrs**	5(17.86)	6(13.04)	0.738
**Male(n,%)**	13(46.43)	19(41.30)	0.666
**EF(%)**	65.82±12.64	43.70±17.55	0.000
**LVDD(cm)**	34.75±5.08	45.27±11.39	0.000
**FS(%)**	32.87±6.31	21.85±8.77	0.000
**Creatinine(umol/L)**	36.28±11.53	31.08±12.62	0.080
**Glucose(mmol/L)**	4.65±0.59	4.85±0.57	0.155
**Total glyceride(mmol/L)**	0.82±0.63	0.86±0.38	0.741
**Total cholesterol(mmol/L)**	4.56±1.10	4.04±0.79	0.020
**HDL cholesterol(mmol/L)**	1.44±0.44	1.36±0.33	0.372
**LDL cholesterol(mmol/L)**	2.71±0.89	2.33±0.73	0.049

Values are mean±SD or n(%). The p values are quoted for the Kruskal-Wallis or chi-square tests for continuous or categorical variables, respectively.
DCM: dilated cardiomyopathy; EF: Ejection Fractions; FS: fractionalshortening; HDL: high-density lipoprotein; LVEDD: left ventricular end-diastolic dimension; LDL: low-density lipoprotein.

Supplementary Table 4. qPCR Primers used for detecting miRNAs expression

Genes	Primer sequence(5’-3’)	Annealing temperature	Length of targets
**U6**	F:5’GCTTCGGCAGCACATATACTAAAAT3’<br>R:5’CGCTTCACGAATTTGCGTGTCAT3’	60	89
**miR-26a-5p**	GSP:5’ GGGTTCAAGTAATCCAGG3’<br>R:5’ GTGCGTGTCGTGGAGTCG3’	60	62
**let-7g-5p**	GSP:5’GGGGGATGAGGTAGTAGTTTGT3’<br>R:5’ GTGCGTGTCGTGGAGTCG3’	60	66
**miR-143-3p**	GSP:5’ GGGATGAGATGAAGCACT3’<br>R:5’ GTGCGTGTCGTGGAGTCG3’	60	62
**miR-126-3p**	GSP:5’ GGGGGCATTATTACTTTTGG3’<br>R:5’GTGCGTGTCGTGGAGTCG3’	60	64
**miR-24-3p**	GSP:5’ GGGTGGCTCAGTTCAGC 3’<br>R:5’GTGCGTGTCGTGGAGTCG3’	60	65
**let-7f-5p**	GSP:5’GGGGTGAGGTAGTAGATTGT3’<br>R:5’TGCGTGTCGTGGAGTC3’	60	63
**let-7i-5p**	GSP:5’TGGGGGTGAGGTAGTAGTTTGT3’<br>R:5’GTGCGTGTCGTGGAGTCG3’	60	66
**miR-142-5p**	GSP:5’GGGGCATAAAGTAGAAAGC3’<br>R:5’CAGTGCGTGTCGTGGAG3’	60	65
**miR-27a-3p**	GSP:5'GGGTTCACAGTGGCTAAG3'<br>R:5'CAGTGCGTGTCGTGGA3'	60	64
**miR-27b-3p**	GSP:5’GGGGGTTCACAGTGGCTAAG3’<br>R:5’GTGCGTGTCGTGGAGTCG3’	60	64
**miR-98-5p**	GSP:5’GGGGGTGAGGTAGTAAGTTGT3'<br>R:5'CAGTGCGTGTCGTGGAGT3'	60	67

Suppelementary Figure 1.

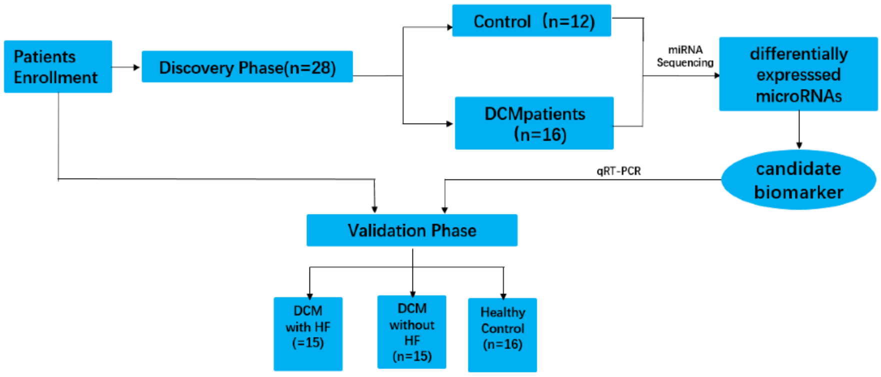

Supplementary Figure 2

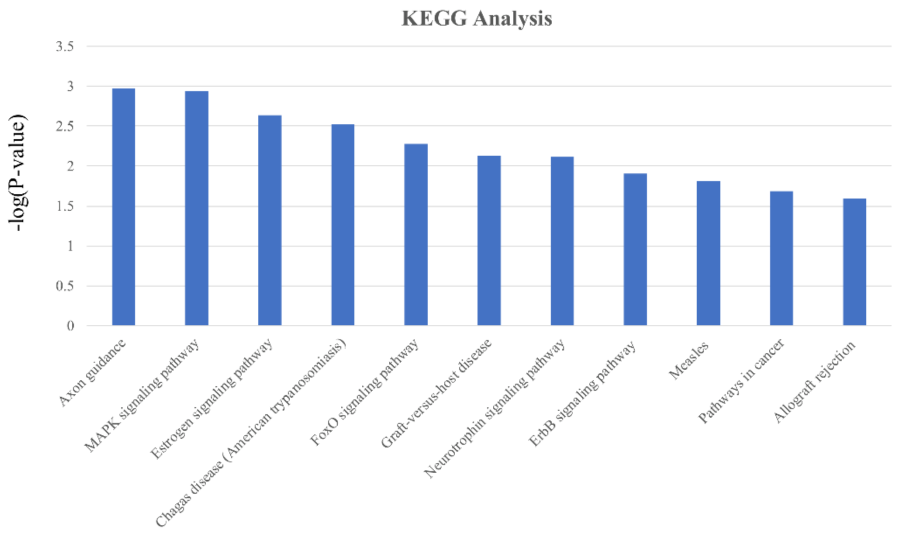

Supplement: Supplementary file 1 — Supplementary Material [file 41598_2017_19138_MOESM1_ESM.pdf]
